# Studying the effects of virtual biodiversity research infrastructures

**DOI:** 10.3897/zookeys.150.2164

**Published:** 2011-11-28

**Authors:** Daphne Duin, Peter van den Besselaar

**Affiliations:** 1 VU-University Amsterdam, Dep. of Organization Sciences. & Network Institute De Boelelaan 1081, 1081 HV, Amsterdam, The Netherlands

**Keywords:** Knowledge creation, users, scientific collaboration, Webscience, impact assessment, biodiversity and taxonomic research infrastructures, virtual communities of practice

## Abstract

The research environment of scholars is increasingly web-based. This makes it urgent to study the effects of moving to the Web on research practices, scholarly output and innovation. We propose a theoretical framework and a methodology to study these effects. In a pilot study, we apply theory and method on an online community in biodiversity research, to demonstrate the feasibility of the approach. We also indicate the practical relevance of this kind of analysis for improving the quality of virtual research environments. In the last section, directions for further research are suggested.

## Introduction

Moving science to the (social) Web has generated excitement for its potential to support knowledge creating activities in research environments (eResearch2020 2010). Core ideas behind social web applications and services are: to make the Web a place for user generated content; to harness the power of crowds; to provide access to data on a large scale; to offer an architecture for participation and to create network effects and openness (Tim O’Reilly in: Anderson 2007 p.14). The social Web, also called Web 2.0, brings people, ideas, tools and information resources together and is in this way a promising means to accelerate scientific developments and to disseminate scientific information for policy and education.

In the field of biodiversity research numerous Web based tools are currently available. The tools facilitate knowledge creation within the global expert community of biodiversity researchers and bioinformaticians. The tools allow users to do collaborative work on the Web. Users can create content, share data and have access to knowledge that was once only available to individual researchers, whether in paper achieves, on stand-alone computers or in difficult to access data systems of their institutions. Several of these kind of tools are supported under the 7^th^ Frame work Programme ViBRANT. A sum of such tools and online services is referred to as Virtual Research Environments (VREs), a cyberinfrastructures or e-infrastructures (Fraser 2005). These concepts are continuously evolving and often used interchangeably. The different terminologies have in common that they comprise digital infrastructures and services which enable research to take place (idem). Even though the expectations on the impact of Web-based science are high, most virtual research environments, being relatively new, struggle with engaging user communities and with the implementation of a sustainable model.

Within the context of a larger trend to move biodiversity to the Web (see also: Global Biodiversity Information Facility GBiF; Encyclopedia of Life EOL; Biodiversity Heritage Library BHL; ViBRANT), we are interested in the effects that the move to the Web has on researchers’ work environment, research practices, scholarly output and the changing needs for support (see also JISC Virtual Research Environment programme). A better understanding of the effects will contribute to: i) a design and management that better fits the needs of the users; ii) improving sustainability; iii) and more generally to research on infrastructure policy.

In this paper we will zoom-in on the questions mentioned above. Our main aim is methodological. We will elaborate a method for studying the effects of web-based biodiversity research infrastructures on scientific collaboration, innovation, and performance. In what follows we will put forward a theoretical framework, discuss empirical data and a methodology - which we think will help in answering the question. To illustrate the possibilities and limitations of the methodology suggested we will discuss empirical data that we collected for a pilot study on one online community of the Scratchpad platform [http://scratchpads.eu/] and conclude with recommendations for further research. Scratchpads are an online platform for collaborative and distributed work in biodiversity research. The Scratchpad environment is currently one of the more established services that is coordinated under the ViBRANT FP7 umbrella and is in the air since 2007.

Here we stressed why it is important to examine the effects of moving science to the Web. In the following paragraph we bring together previous research on the organisation of knowledge creation and discuss how we think we can use these findings in our own work.

## Knowledge creation

Knowledge creation is at the heart of the academic profession. Influencing the creation of new knowledge is a challenge for organisations as knowledge flourishes best when it is enabled, not managed (Sveiby 2001). Key concepts for understanding knowledge creation are “implicit knowledge” and “explicit knowledge” as put forward by Nonaka et al. (1994, 1995). Implicit knowledge is experience based and context specific knowledge that cannot be expressed in words, sentences, numbers or formulas. This also includes cognitive skills such as beliefs, images, intuition and mental models as well as technical skills such as craft and knowhow. Explicit knowledge is codified, general knowledge that can be expressed in words, sentences, numbers or formulas. It includes theoretical approaches, problem solving, manuals and databases (Nonaka 1997). According to the authors, the answer to mobilisation and creation of knowledge is to enable interaction and the exchange of implicit (tacit) and explicit (codified) knowledge (Nonaka et al. 2000). Woo et al. (2003) and Herschel et al. (2001) emphasise that converting implicit knowledge to explicit knowledge is often seen a problematic task, labour intensive and expensive. One solution to overcome this problem is the creation of Communities of Practice (CoP). These CoPs bring together knowledgeable experts to work on complex problems (Andriessen 2005; Wenger 1998). This relates also to Sveiby’s (2001) observation that implicit knowledge is best kept in knowledgeable people and is achieved by making knowledgeable people communicate. According to him: “knowledge shared is knowledge doubled” (p. 347). McFayden et al. (2009) add to this the importance of combining diverse and overlapping knowledge inputs between exchange partners for the creation of new knowledge. Overlapping knowledge allows for greater specialisation and support in CoPs because a common knowledge base (e.g. mental frames, shared knowhow) eases communication (Demsetz 1991). On the other hand, heterogeneous or sparse networks provide more opportunities to secure access to new information and diverse perspectives (Burt 2001). In other words, a CoP needs a common basis of implicit and explicit knowledge for good communication flows and stability but also diversity in order to be innovative and flexible.

Next to the contributions from knowledge management studies on innovation, also social network studies have also contributed important insights to our understanding of the conditions and constrains for knowledge creation. Scientists, like other professionals, bring more to work than skills and experience, “they also bring the assets they can procure through their social networks” (Gargiulo et al. 2000: p. 183). This is often referred to as “social capital” (Bourdieu 1980; Coleman 1988 in: Gargiulo et al. 2000 p. 183; Burt 2001, 2007). Burt demonstrates that “compensation, positive performance evaluations, promotions, and good ideas are disproportionately in the hands of people whose networks span structural holes” (2004, p. 349). Structural holes are non-redundant connections between actors in a network. In other words, structural holes are ties to people that are themselves not connected. People in an organisation who connect not connected groups are called “brokers”.

**Figure 1.**
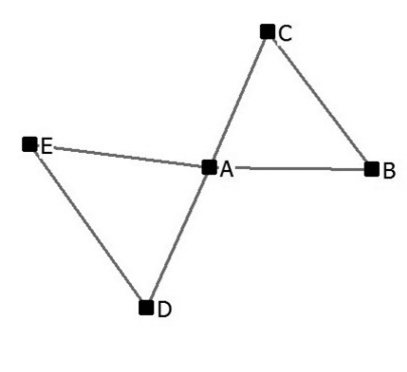
Brokerage and structural holes.

Social network studies make use of sociograms to support their analysis. These are graphic representations of social links that a person has. Figure 1 is an example of a sociogram of structural holes that are linked by of one actor in a network, represented by node A at the centre of the graph.

The nodes are actors, the ties their connections. Actor A is a broker in this network because connects two groups that are otherwise unconnected (spans structural holes).

Social network studies show that brokers are valuable individuals for organisations. Brokers are people who have the capabilities to “”translate, coordinate and align between different perspectives (…) and address conflicting interest” (Wenger 1998: p. 109). Moreover brokers are more likely to express new ideas and to have them judged valuable (Burt 2004). This idea of “selection and synthesis across structural holes and between groups is not new” (Burt 2004: p. 350). Hence, most structural holes studies were carried out among local based workers (e.g assembly line workers of the same factory). What we aim to study is how this functions in online (distributed) research communities.

Virtual research environments like Scratchpads aim not to replace existing data and communication systems but, rather offer additional ways of working with existing facilities. They add an additional organisational and network layer to the traditional work environment of a researcher. Researchers already participate in multiple professional and personal networks such as: at the level of their department; the institutions; national/international projects: alumni networks; advisory boards etc. Becoming a member of an online work group would add another network layer to their organisation of work. We would like to argue that to be able to study the effects of moving biodiversity online we have to take into consideration already existing structures of the researchers work environment and investigate to what extent these change when a new way of working is adapted. Hence, instead of looking at uniplex networks a study of multiplex networks will be helpful in getting a deeper understanding of how the introduction of a new network layer might change exiting organisational structures. As Lee et al. demonstrate “multiplex networks involve multiple relations that create multiple ties in one network have been shown to influence the formation or dissolution of ties in other networks” (2011, p. 759).

Today, online networks are important vehicles for knowledge sharing and learning in the workplace (Ardichvili 2008). The expectation is that the social Web provides enabling conditions for knowledge creation as mentioned above. The social Web overcomes a number of barriers for knowledge exchange and interaction - by giving distributed communities the tools to control the level of openness of their communication and tools to simulate a face-to-face setting with help of online instruction videos, VOIP, document- /image- / biography- sharing tools, forums and other layouts of online communities. Triggered by the developments of Web 2.0 tools, the playing field of CoPs moves to the Web, which turns them into Virtual Communities of Practice (Samarah et al. 2008). The claims about the usefulness of Web 2.0 tools for knowledge creation are often made. However, there is a surprising dearth of empirical studies that show the impact of Web 2.0 tools on knowledge creation in virtual communities, with the exception of work by Samarah et al. (2008).

In summary, knowledge flourishes when knowledgeable people are brought together and interact. Especially the exchange of different but partly overlapping knowledge enables the creation of new knowledge within expert communities. Another important enabling condition that arises from the literature is the amount of social capital of individual actors as well as social capital kept within collective working groups (teams, labs, departments). The open question to be studied is whether Web 2.0 tools, such as Scratchpads, do provide these conditions. This is something to be studied.

In this paper we will discuss a pilot study that examines the possibilities of a social network approach to study co-authorship and Scratchpad membership. But before we come to discuss our pilot study we will investigate the challenges of studying online social settings. Web data are still a relatively new empirical data source in the social sciences and there is some debate on how to collect and interpret data sets collected from the Web. In the following paragraph we will discuss some of the pros en cons of the use of web data to study organisation(s).

## Virtual communities of practice

The Web has become a major medium for communication in science. ViBRANT products and services, currently being developed under the 7^th^ Frame Work programme, mirror a trend within biodiversity research moving science to the Web. In this paper we concentrate on one of the products of ViBRANT, the Scratchpads. Scratchpads are an online platform for biodiversity research where virtual communities of academic experts link remote resources together (people, biographies, images) and offer an environment for learning and knowledge creation that before was only possible in geographical proximity (Smith et al. 2009). The platform has today a global user community of > 3000 people, which is steadily growing. The communities are created around different biodiversity research topics, such as around a particular group of organisms, around a project, or bioinformatics topics. Scratchpad communities are managed by individual researchers that apply for a site and can invite /or make it open to fellow researchers to register and participate in content sharing and analysis. Some sites are communities-of-one, other sites have more than 200 registered users. Also the level of activity among users of one Scratchpad may vary significantly. The content creation of some sites is the effort of a single researcher, while the other users of the site take a more “passive” role. For other Scratchpad sites the whole community is actively engaged in the creation of content. What all users have in common is that at one point they decide to register as a user of a community. They either saw an interest in connecting to the other users or to the content of the site, they identified with the people, the content or with both.

From a previous study that we did among the users we know that Scratchpads are used among biodiversity researchers mainly: to disseminate research results; to share data; to collaborate in the writing of project proposals and papers; and for preparing meetings (Smith et al. 2010 p.4). In the field of organisational knowledge creation Scratchpads can be coined Virtual Communities of Practice.

*Virtual Communities of Practice are a type of knowledge based social network whose members rely primarily on networked ICT’s in order to 1) discuss problems and issues associated with their day to day activities 2) collaborate on projects 3) share documents, solutions or good and bad practices, plan for face to face meetings or continue face to face relationships and work beyond face-to-face events (Anandarajan and Anandarajan 2010, p.154)*

The move of science to the Web leads to new questions regarding the impact of the online environment on scholars’ behaviour, relations, and scholarly output. It also provides us with new types of data and methodologies. The Web is constituted by a myriad of socio-technical interactions which often leave digital traces. Users of the Web leave digital footprints of their behavior and network relations. Their footprints can be found in web server log file data or in the information that is stored on institutional web pages and social network sites. Consequently “it forms an interesting, modern site for research ethnography” (Beaulieu 2005, p. 183) and for quantitative studies of these digital traces (cf. Thelwall 2010). Today also offline activities can be studied from the Web as personal information about researchers’ work is disseminated widely online, in publication databases, conference websites and sharing tools for presentations and images, just to name some examples.

The use of such data sets for social research, like the digital footprints of researcher’s online activity, has several advantages. Firstly, the scale on which we can do research becomes much larger, as one can collect large datasets covering the actions of many users and over long periods of time and geographical distances. Secondly, research using such data is unobtrusive as the actors under study are not interrupted in their work by data collection activities. Thirdly, the data are observational, and not based on opinions only, such as in surveys and interviews. Fourthly, costs are potentially lower, as web data can be collected from behind a desk and are often freely available (cf. Johns et al. 2004). Hine (2005) describes the use of the Internet data for social research as a trend where excitement and anxiety come together. Some of the advantages are mentioned above. The disadvantages of the use of web data relate to lower responses rates of online surveys; non-representative of the sample, a decreasing quality of the data, and privacy issues. As a consequence, it is often argued that (secondary) web data is best used in combination with other, primary data, to control for issues such as representativeness (Buckman 2006). Something we plan to do in future research. In the next section we discuss the methods that we used for analyzing web data.

## Pilot study, data and methods

As argued, the creation of new knowledge can be enabled by bringing a variety of knowledgeable people together in an environment that facilitates the interaction and exchange of heterogeneous and overlapping knowledge inputs. We carried out a pilot study on one Scratchpad community to explore this question and test our approach. The research questions are: 1) to what extent do Scratchpads connect people that were not connected before (as co-author)? 2) To what extent do Scratchpads create new links between different bodies of knowledge (structural holes) and reinforce existing links?

For both questions we build on ideas and techniques stemming from bibliometrics (cf. Glänzel 2002) and Social Network Analysis (cf. Wasserman and Faust 1994). In the literature section above we explained why Social Network Analysis offers a useful framework. In order to answer the first question we compare the offline, traditional collaborative network connections of the Scratchpad users, their co-author relations, with Scratchpad membership. In other words, we are interest to know who connects to whom because of membership who was not connected before by co-author relations. Or, were all users already connected before they joined and is the Scratchpad only a different media to continue to work with people one already used to collaborate with? Co-author relations are valuable measure for academic collaboration but should be handled with care (Glänzel 2002). Also, co-authorship is certainly not the only form of collaboration in science. People are part of multiplex social networks (e.g department, institutions, editorial boards). Each network has its own type of interactions (drinking coffee, talking in meetings, peer reviewing on the same journal). The combined number and type of network connections and interactions has an effect on someone’s social capital. Sometimes networks overlap, you meet the same people in different settings taking on different roles. But sometimes new networks do not overlap and fill “missing links” in one’s social capital. In our data example we stack two networks on top each other: the co-author network and the Scratchpad membership network, and study to what extent the Scratchpad membership connects researchers that were not already connected by co-authorship ties.

The second question deals with the potential of the Scratchpad community to create new knowledge (span structural holes) and create favorable conditions to continue to exist over a long period of time (redundancy). For this question the Scratchpad community is taken as an analogy for a research team where every member brings in social capital in the form of their co-author network. This time we did not look at the co-author relations among the 11 members but to what extent their ego, co-author relations overlap. Do the Scratchpad members co-author with the same peers, or do they bring in their personal, unique co-author contacts?

Our case is a single Scratchpad which we give here the fictional name Livingcreatures.info. User registration coming from automated bots, so called spam-signs ups, were excluded from the analysis. The member list that we used included members’ personal details such as their affiliation and was used as the starting point of studying co-author relations. For each member we collected their publications over a period of 10 years, preceding their online collaboration in Livingcreatures.info. Publications were searched for and downloaded from the ISI Web of Science database and combined with publications from Google Scholar (using Publish or Perish). The Web of Science is a much more structured database, with for instance better name ambiguity filters than Publish or Perish. However the combination of both was thought important as biodiversity research is underrepresented in the Web of Science (cf. Krell 2002). Therefore we needed to complement this with publication information from additional data sources. Publish or Perish uses Google Scholar data, with a much wider coverage. The resulting publication lists cover journal articles, books chapters, series and peer reviewed and non-peer reviewed papers. In the next step we retrieved the co-author relations of each Scratchpad member and this enables us to study to what extent the co-author relations of the members overlap. This indicates the degree of differences and similarities between the types of knowledge represented in Livingcreatures.info.

The Scratchpad under study was launched early 2011. As for August 2011, this Scratchpad has 11 registered members, all male. Ten of the members were in the period of our analysis affiliated to one of the natural history institutions in the world, number 11 is mentioned in the acknowledgements as a private taxonomic specialist. Their institutional addresses are located in five different continents (3 in Europe, 3 in Asia, 2 in Africa, 1 in South America, 1 in Oceania). Together, these 11 Scratchpad members have 187 co-authors (including inter-group relations) with whom they collaborated in the period from 2001-2010. Table 1 gives the breakdown of the publications of the group. Together they contributed to 135 publications in ten years. Four Scratchpad members have no co-author relations. The information that we found during our web search suggests that this may be explained by individual characteristics. From the web data we learnt that two of them are early career researchers, number three is in a non-research position in a research institute, and number four is a volunteer researcher. Table 1 shows the details about the publications and co-authors of the 11 members.

We collected and analyzed the co-author data of the members of one Scratchpad and applied a social network approach to the data in order to get a better understanding of the effects of moving biodiversity research to the Web. In the next paragraph we discuss the results of the analysis.

**Table 1.** Scratchpad members^†^, number of papers and their co-authors. ( 2001-2010).

Scratchpad members	number of publications	number of unique co-authors	number papers with one author	number of papers with co-authors	number papers with > 2 authors	max number of co-authors on 1 paper
Group total	135	180	18	117	80	-
member 1	30	66	3	27	18	19
member 2	18	40	4	14	13	9
member 3	0	0	0	0	0	0
member 4	16	21	1	15	14	6
member 5	0	0	0	0	0	0
member 6	1	3	0	1	1	2
member 7	17	52	1	16	11	16
member 8	70	40	9	61	36	4
member 9	0	0	0	0	0	0
member 10	0	0	0	0	0	0
member 11	3	4	0	7	1	2

^†^ Members from 1 Scratchpad site. Group total is not the sum of the cells, several members have collaborated on the same publication

## First results

We operationalised the two research questions in the following way: Does Scratchpad membership: i) connect people that were otherwise not connected; ii) provide network conditions that are beneficial for the creation of new knowledge and conditions for stability, that is, does the Scratchpad link researchers from different but not too different fields?

### Do Scratchpad connect?

**Figure 2.**
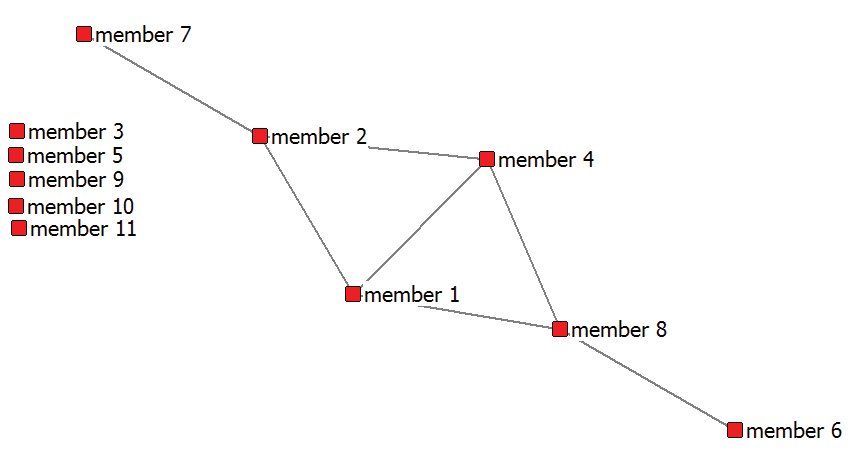
Graph of co-author ties^†^ between the members of the Scratchpad Livingcreatures.info^‡^. (2001-2010).^†^ Data sources: Web of Science and Publish or Perish. ^‡^ For privacy reasons we use a fictional name.

We used UCINET6 (Borgatti et al. 2002), a software tool for social network analysis, to construct the co-author matrices and to visualise the co-author relations within the Scratchpad community and with authors outside the community. Figure 2 is a visualisation of co-author ties between Scratchpad members (inter-group relations). The graph is based on a (symmetrical) adjacency matrix with 11rows and 11 columns. If member 1 has published with member 2 the cell contains a 1 if they did not publish together the cell entry is 0. The nodes represent the 11 Scratchpad members, the lines between the nodes their co-author relations.

What does the network show? The graph shows that six of the members are connected through co-author relations. They do not form a dense clique as they do not connect all co-authors with each other. Of these six, four have published with three others in the group, the two members positioned at the tips of the graph have published with only one other group member. The four members (1, 2, 4, 8) in the center of the graph (with each three links) already were acquainted with one of the co-authors in the co-author network of their fellow Scratchpad member. On the other hand the two members in the tips seem more “peripheral players” in *this* network.

**Figure 3.**
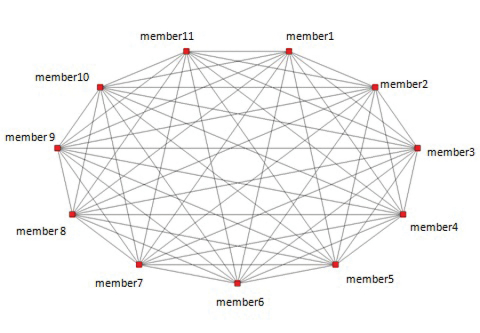
Graph of membership ties among Scratchpad members Livingcreatures.org

Figure 2 also shows that the Scratchpad connects the five isolated members (3,5,9,10,11) with each other and with the members that already co-authored before they joined the Scratchpad. In other words, the five isolates are each connected to 10 potential “new” peers. If we compute in a similar way a sociogram of the Scratchpad this would look as followed, see figure 3. In the Scratchpad the 11 members are all linked to each other by *membership* of the same community. Note that a membership tie is different from as a co-author tie. A membership tie refers to sharing common interests and resources, a co-author tie refers to jointly producing a publication.

**Figure 4.**
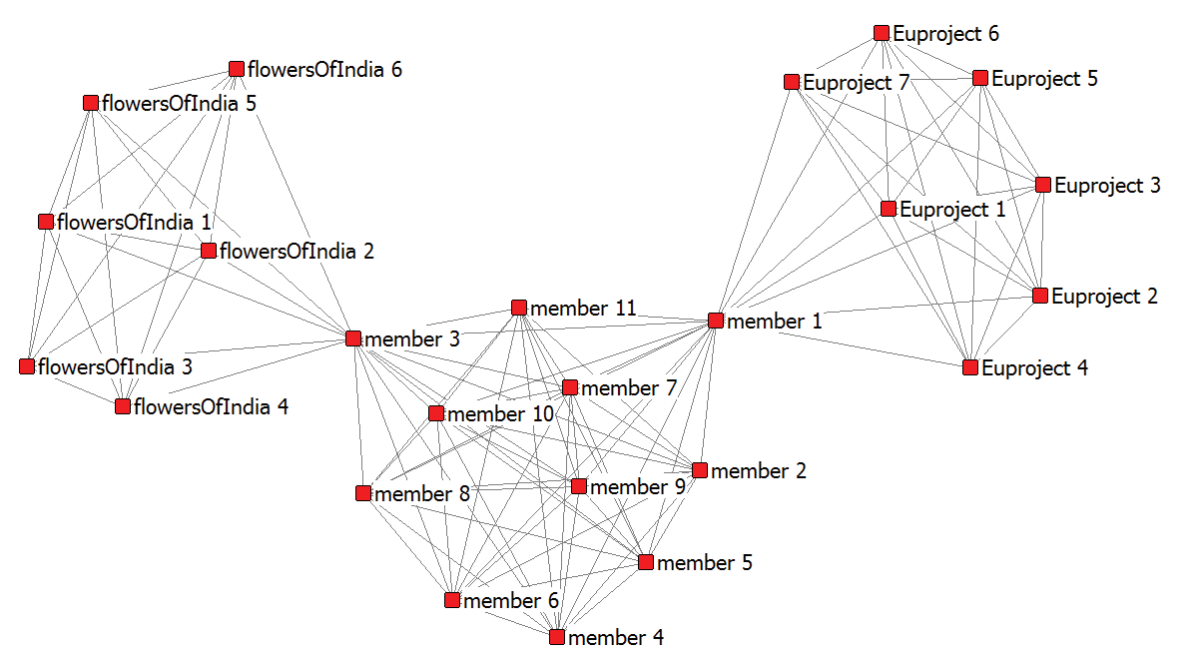
Livingcreatures.info and ties with two other Scratchpads. This graph shows a fictional situation.

We studied the network of a particular Scratchpad community in isolation, not taking into consideration a possible overlap between different Scratchpads (currently more than 200 communities are active). Figure 3 looks trivial but serves to demonstrate the different structure as opposed to the co-author network (Fig. 2). Figure 4 is a fictional example of a network structure of how the Scratchpad under study (this one is real, only has a fictional name!) might be embedded in a lager structure of Scratchpad communities. In this fictional example two members of Livingcreatures.info are also members in other Scratchpads, one around an EU project the other one of Flowers of India. This is a graph of a fictional situation to show possible complexity in the larger Scratchpad structure.

In the next step, we extend the co-author network of the members with the co-author relations from outside the Scratchpad. The question is whether including these links changes the network topology, and whether the isolates are still isolates in the larger co-author network.

### Overlapping and diverse knowledge

**Figure 5.**
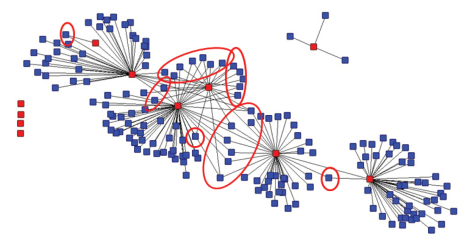
Graph of co-author ties^†^ between the members of the Scratchpadlivingcreatures.info^‡^. (2001-2010). ^†^ Data sources: Web of Science and Publish or Perish. ^‡^ For privacy reasons we use a fictional name.

The literature discussed above concludes that a main enabling condition for knowledge creation and innovation is the prevalence of a mix of overlapping and diverse types of knowledge inputs that are exchanged. Scratchpads do so, if the membership is scholarly heterogeneous. Another conclusion is that if stability is important, a certain level of redundancy in the network is important (Gargiulo et al. 2000). Figure 5 is the visualization of these connections. This graph is based on a symmetrical 192 × 192 matrix representing the 192 authors and their “external co-author relations”.

In red we still see the Scratchpad members. The layout is similar to Figure 2 to facilitate comparison. In blue we have the authors that are not in the Scratchpad. The red circles indicate those non-members that co-author with more than one Scratchpad member. Adding the external co-authors does not change much how the Scratchpad members are linked. The four core members have several indirect relations, in contrast to the more marginal members who lack these indirect links. Member 9 has his own small network. Adding the external authors did not link him to the large component. The other four members are the isolates, nodes without any co-author relations with other nodes.

Table 2 shows the number of the shared co-authors for each of the members. Note that the co-author relations between Scratchpad members are not included. Seven Scratchpad members have co-authors who are also co-authors of other members. The number of ‘overlapping connections’ (redundancy) ranges between 1 (for members 6, 7, and 11) and 3 (members 1, 2, 4, and 8).

Table 2 shows us that there are only a few shared co-authors and most of the 180 co-authors (see Table 1) are not shared by the Scratchpad members. The average number shared co-authors do not differ much among them. Total number of redundant connections that are brought in by the members is much lower than their contribution to bringing in new co-authors and so span structural holes. Redundancy as mentioned here refers to the Scratchpad level, meaning that overlapping co-authorships are redundant for the social capital of the Scratchpad community as a whole. At the level of the individual actor however the connection might have an added value. Redundancy at the network level will rise when Scratchpad members increase their collaboration with the same co-authors from outside the Scratchpad or when co-authors would decide to join the Scratchpad. Redundancy in the Scratchpad network will secure “access” to a specific co-author or group of authors. The goals that the Scratchpad members have set will define if more redundancy (more overlapping connections) at the network level is useful for the group.

The shared co-authors are also interesting because they are part of the professional networks of several of the Scratchpad members, and therefore they may have an interest to join the Scratchpad. The network suggests that they would contribute to intensifying interaction and to the exchange of knowledge. Adding them to the network will add a second type of redundancy and therefore stability. However, from an innovation point of view, adding redundant actors to a network is wasted energy.

We conclude that the Scratchpad under study is a rather globally distributed Virtual Community of Practice in biodiversity research, some members have a long record of publications and others have a much shorter list (see Table 1), possibly because they just starting their academic career. Also from Table 1 we conclude that the Scratchpad members of this community have a collaborative attitude. Their publication behavior demonstrates that they are used to collaborate with several authors on one paper, running from two authors up to 19 authors on a paper. When comparing co-author relations and Scratchpad membership we see that signing up for the Scratchpad has created new ties for every one of the 11 members, though some gained more new connections than others. Scratchpad members do share co-authors from outside with their fellow Scratchpad members, showing that the two knowledge networks (author network, Scratchpad network) partially overlap. However, most co-authors of every Scratchpad member are new to the other members, suggesting that members bring in not only similar but also different knowledge sources and skills. Depending on the goal of the Scratchpad the members could try to increase either the overlapping - either the diverse types of knowledge inputs at the network level (redundancy versus spanning structural holes).

Of course, co-author ties and Scratchpad networks only form two of many types of networks of researchers. Other networks (e.g. based on organisation membership, committee membership etc.), may change the network configuration, and therefore may show a different role and effect of Scratchpads in the total network of biodiversity research. This is something to consider in future investigation. A second issue that needs further research is what Scratchpad members actually do in the Scratchpad, as this may teach us about the nature of the Scratchpad relations.

**Table 2.** Scratchpad members and number of co-authors they share with fellow members. (2001-2010).

Scratchpad members Livingcreatures.info	Number co-authors † shared with fellow members
member1	3
member2	3
member3	0
member4	3
member5	0
member6	1
member7	1
member8	3
member9	0
member10	0
member11	1

^†^ Data sources: Web of Science and Publish or Perish.

## Discussion

This paper aimed to explore what theoretical framework, method and data can be used to study the effects of the increasing role of web-based research environments on the practice, innovation and performance of biodiversity researchers.

We used the rich body of theories on organisational dimensions of knowledge creation, which suggests enabling conditions for knowledge production and innovation. The design of Scratchpads is partly based on the criteria in “bringing together knowledgeable people in communities of practice”. Social network theory teaches us how to study and assess the network configuration of these knowledgeable people, as the patterns of links determines the added value for individual actors as well as for the network as a whole, in terms of social capital, knowledge creating power and stability. The concept of multiplex networks reflects that scientists work in a ‘multi layered’ research environment: e-scientists are active in a variety of professional networks in and outside their organisation, real and virtual.

As science moves to the Web, the behavioral footprints of scientific work practice are more and more available as (secondary) web data. Web data are an inexpensive way to ‘observe’ the behavior of large groups of people. From Buckman (2006) we took that the there is a challenge to compile a representative data set from the Web and that therefore the data has to be controlled for with use of a primary data set.

From our pilot study we learnt that through computing of relatively simple graphs, we get a better understanding of the effects of Scratchpad membership on scholarly networks. It enables us to compare characteristics of Scratchpad networks with e.g. co-authors. Our analysis suggests that Scratchpads do create links between researchers that do not exist in the co-author network, and therefore fill structural holes in the network. This is one of the enabling conditions for the creation of new knowledge.

Analyzing the number of shared co-authors among members of Scratchpad Livingcreatures.info indicates that the members form a loosely collaborating group of researchers. However, if we also take into consideration the collaboration between the members, the network seems denser. Some Scratchpad members were already collaborating before joining the Scratchpad, however most co-author relations are from outside the Scratchpad community. In other works, the Scratchpad partly reinforces already existing relations, but also creates new links for those members that were not yet included in the co-author network.

Our pilot study shows that the selected approach is promising. In the next phase we will extend the study in several ways. Firstly, we used data on only one Scratchpad. We plan to repeat the analysis for a large set of Scratchpads, which will enable us to test whether the level of variety correlates with knowledge production and innovation, as the theory suggests. Secondly, in the current pilot study we treated all co-author relations as having the same importance, which does not well reflect real world relationships. This is also something to take into account in future research. Thirdly, we used only two different networks of the researchers (Scratchpads; co-authorships) while neglecting many others, such as organisational proximity, professor-student relations, project membership, and scientific specialisation. In order to get the full picture of the role of Scratchpads in scholarly networks, the analysis should be extended with the kind of networks mentioned. Fourthly, it is crucial to compare Scratchpad members with non-members, in order to test if changes in research practice and performance of members are different from eventual changes in the field at large. Finally, the Scratchpad we studied in this paper was launched in 2011. In order to assess the effects of the deployment of virtual environments, we suggest using a longitudinal research approach: have co-author networks and the thematic orientation of Scratchpads users changed over time, and is this change different from other researchers in the field?

These questions are not only theoretical relevant, but may also be useful in the practice of organising Scratchpads and other virtual research environments. It may also help to identify potential interesting new Scratchpad members that might be actively invited to participate. Moreover, these lines of research could contribute to sustain user engagement and to general research infrastructure policy.
